# Editorial: A Message from the Editor-in-Chief

**DOI:** 10.1523/ENEURO.0023-17.2017

**Published:** 2017-02-03

**Authors:** Christophe Bernard

Dear friends and colleagues,

It is a great pleasure to tell you that *eNeuro* is very successful. Since our launch in November 2014, we have received over 500 submissions and published over 300 papers. In our second full year, we published 210 manuscripts, which more than doubled our 86 publications in 2015. And our website visits have also doubled from 2015 and continue to grow each month. Despite the lack of impact factor (the first one will be calculated this coming year), more researchers are relying on us to publish their results. This vote of confidence not only shows that *eNeuro* succeeds in publishing great science, but also that our publishing model satisfies all actors: authors, reviewers, and reviewing editors.

In 2016, we increased the number of reviewing editors to cover more neuroscience disciplines. Our goal is to cover the growing and evolving breadth of the field. I would welcome any suggestion regarding missing topics. You may have noticed that we started actively using our social media platforms to promote some research articles, along with other outreach activities, which help to promote the journal and increase the reach of its publications. There have also been some great commentaries published this year: the Science Educator Award commentary and the Sci Rigor series along with homepage videos that provide insights on publishing in *eNeuro*.

But let us look at the future. We are working hard to bring even more innovations in 2017.

∙ **Acknowledging the best reviewers on the home page**. The reviewing process is an integral part of scientific research. It must be properly acknowledged. Note that since 2016 reviewers can update their ORCID account with the number of reviews they perform for both SfN Journals.

∙ **Educating investigators at an early stage of their career to perform a review is an important mission**. The first webinar we organized was very successful. SfN members can access it here. We are going to organize many others, focusing on specific topics or themes in Neuroscience. Stay tuned for the next one, which will be on computational neuroscience. As before, attendants will be given access to the first submitted version of the paper before the webinar to work on it as reviewers. During the webinar, we will see how the review process was performed during the consultation process, with the do and not-do when reviewing a paper.

∙ **Working on implementing video abstracts**. Authors will be able to upload, if they wish, a video version of the published story for a better outreach to the community.

∙ **Sharing author and editor experiences.** More videos are coming to provide the benefits of consultations with reviewers and the experiences of authors submitting to *eNeuro*.

Thanks to you, *eNeuro* is still on the rise, both in terms of publishing great science and in terms of changing the landscape of the publishing world.

2017 will be even better!

Cheers,

Christophe Bernard

